# Supplementary material for: Non-universal current flow near the metal-insulator transition in an oxide interface
Source: Nat Commun. 2021 Jun 3;12:3311. doi: 10.1038/s41467-021-23393-5 (PMC8175561; doi:10.1038/s41467-021-23393-5)
Supplement: Supplementary file 1 — Supplementary Information [file 41467_2021_23393_MOESM1_ESM.pdf]

# Supplementary Information for
# Non-universal current flow near the metal-insulator transition in an oxide interface


Eylon Persky[1], Naor Vardi[1], Ana Mafalda R.V.L. Monteiro[2], Thierry C. van Thiel[2], Hyeok Yoon[3,4], Yanwu Xie[3,4,5], Benoît Fauqué[6], Andrea D. Caviglia[2], Harold Y. Hwang[3,4], Kamran Behnia[7], Jonathan Ruhman[1], and Beena Kalisky[1]*.

1. Department of Physics and Institute of Nanotechnology and Advanced Materials, Bar-Ilan University, Ramat Gan, Israel.
2. Kavli Institute of Nanoscience, Delft University of Technology, Delft, The Netherlands.
3. Geballe Laboratory for Advanced Materials, Department of Applied Physics, Stanford University, Stanford, California, USA.
4. Stanford Institute for Materials and Energy Sciences, SLAC National Accelerator Laboratory, Menlo Park, California, USA.
5. Department of Physics, Zhejiang University, Hangzhou, China.
6. JEIP, USR 3573 CNRS, Collège de France, PSL Research University, Paris, France.
7. Laboratoire Physique et Etude de Matériaux (CNRS-Sorbonne Université), ESPCI Paris, PSL Research University, Paris, France.

*beena@biu.ac.il

## Supplementary Note 1: Sample growth conditions

In this study, we imaged devices from three LAO/STO sample. LAO films were grown on a $TiO_2$ terminated STO substrate using pulsed laser deposition, and the samples were annealed before and after growth in an oxygen environment[1]. Supplementary Table 1 summarizes the growth conditions (oxygen partial pressure, temperature and duration) for each of the growth stages.

| ID | LAO thickness (u.c) | Pre-anneal | | | Growth | | Post-anneal | | |
|---|---|---|---|---|---|---|---|---|---|
| | | T (°C) | P (mbar) | t (min) | T (°C) | P (mbar) | T (°C) | P (mbar) | t (min) |
| C1 | 12 | 840 | $6.00\times10^{-5}$ | 60 | 840 | $6.00\times10^{-5}$ | 600 | 300 | 60 |
| C2 | 12 | 770 | $6.00\times10^{-5}$ | 60 | 770 | $6.00\times10^{-5}$ | 600 | 300 | 60 |
| C3 | 12 | 840 | $6.00\times10^{-5}$ | 60 | 840 | $6.00\times10^{-5}$ | 600 | 300 | 60 |
| H1 | 10 | 950 | $6.67\times10^{-6}$ | 30 | 800 | $1.33\times10^{-5}$ | 600 | 200 | 60 |

**Supplementary Table 1.** Temperature, oxygen partial pressure, and duration of each growth step.

## Supplementary Note 2: Current reconstruction

For a two dimensional (2D) current distribution, it is possible to reconstruct the current density, $\mathbf{J}(x,y) = J_x(x,y)\,\hat{\mathbf{x}} + J_y(x,y)\,\hat{\mathbf{y}}$, from the magnetic flux data performed by the SQUID. The magnetic field generated by a current density $\mathbf{J}(x,y)$ is given by the Biot-Savart law,

$$B_z(\mathbf{r}) = \frac{\mu_0 d}{4\pi} \int dx' \int dy' \frac{\mathbf{J}(\mathbf{r}') \times (\mathbf{r} - \mathbf{r}')}{|\mathbf{r} - \mathbf{r}'|^3} \cdot \hat{\mathbf{z}}, \tag{1}$$

where $B_z$ is the out-of-plane ($\hat{\mathbf{z}}$) component of the magnetic field, and $d$ is the thickness of the conducting layer. Interpreting equation (1) as a convolution integral, we use Fourier analysis to deconvolve the kernel function from the magnetic field measurements, recovering the current distribution. Further details about implementation of this technique to scanning SQUID data appear elsewhere[2,3]. Figure S1 demonstrates how the qualitative features of the data can be clearly observed in the magnetic flux (raw) data.

Figure S1a shows a raw magnetic flux image of a representative LAO/STO device. The raw data feature long-scale field gradients, in accordance with the Biot-Savart law for a homogeneous current flow, as well as shorter-scale modulations, corresponding to local changes in the current density. As an alternative way to visualize the local modulations, Figure S1b shows the magnetic flux data, after the application of a high-pass filter (allowing spatial frequencies ≥ 0.4 $\mu m^{-1}$). The resulting image is proportional to the gradient of the current density, clearly identifying the edges of the device and the local modulations due to defects, while suppressing the homogeneous part of the current flow. Dipole-like features in the filtered image correspond to local reductions of the current density: the current avoids an area with reduced conductivity, increasing the flow around it. The elongated modulations correspond to stripes with enhanced current flow. The same features are directly visible in the current density magnitude image (Figure S1c). The line cuts in Figure S1d demonstrate how locally reduced, locally enhanced, and homogeneous current flow appear in the magnetic flux data, and how the current reconstruction quantifies this effect.

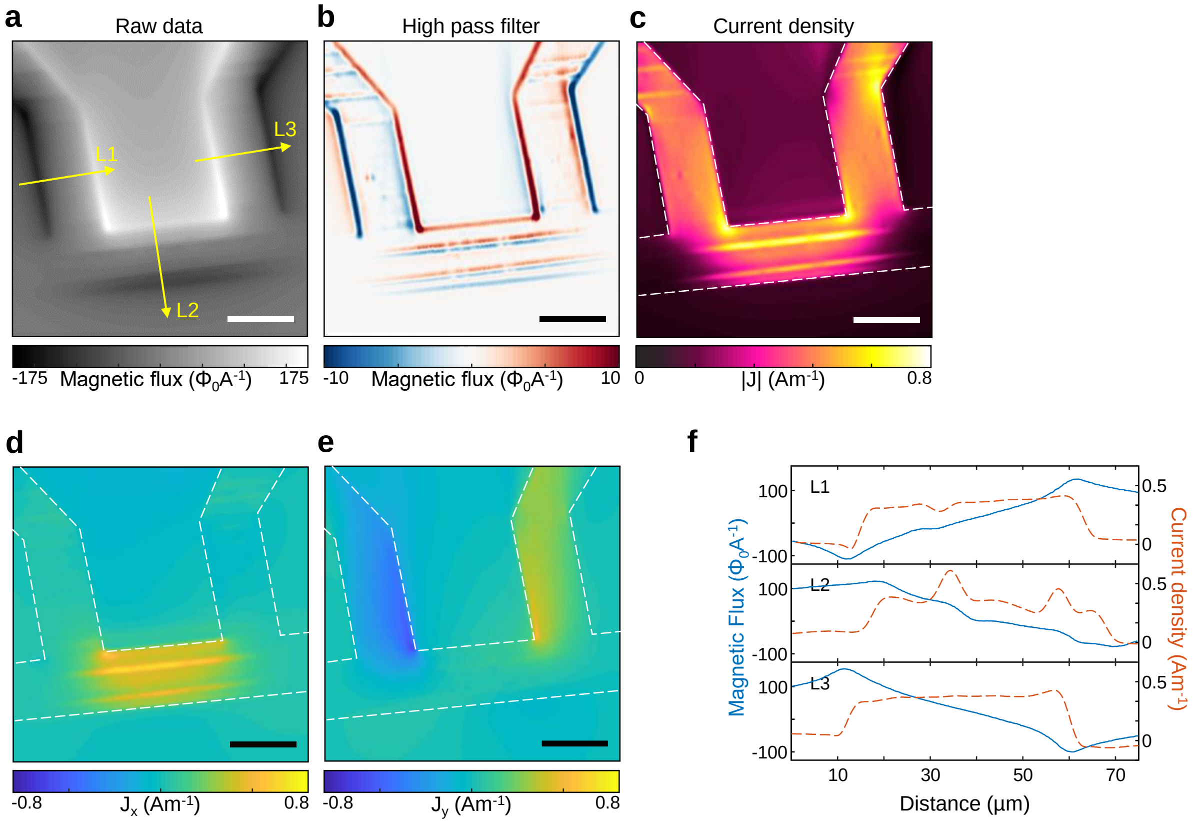


**Figure S1. Current reconstruction. a,** Raw magnetic flux data (normalized by applied current) from sample C2, due to a current of 20 µA flowing in a device 50 µm wide. $\Phi_0 = h/2e$ is the magnetic flux quantum. **b,** Magnetic flux data after application of a high-pass filter, enhancing the local modulations of the current flow. **c,** The reconstructed current density (magnitude) map. Scale bars, 50 µm. **d, e** Current density components in the x (d) and y (e) directions. **f,** Line cuts taken along the yellow lines in a, showing the magnetic flux profile (solid lines) and the reconstructed current density (dashed lines). Lines L1 and L2 correspond to local reduction and local enhancement of current flow, respectively, while line L3 corresponds to a homogeneous current distribution.

## Supplementary Note 3: Transport properties near the MIT

Unlike other semiconductor-based 2DEGs[4], the resistance of LAO/STO devices does not show strong temperature dependence in the insulating phase[5–7]. The gate tunable MIT is characterized by a sharp increase in resistivity, over a narrow range of $V_G$. The high resistance state is often accompanied by non-linear current-voltage (IV) characteristics[5]. Our samples showed behavior consistent with such a transition. Figure S2 shows transport measurements on devices from samples C1 and C3. The zero-bias resistance of the first (second) device abruptly increased as we lowered $V_G$ below -40 V (-8 V). In both devices, highly non-linear IV curves appeared around these voltages. Although the details vary between the samples, the qualitative aspects of the transition are similar, and a clear distinction between the insulating and metallic phases can be drawn.

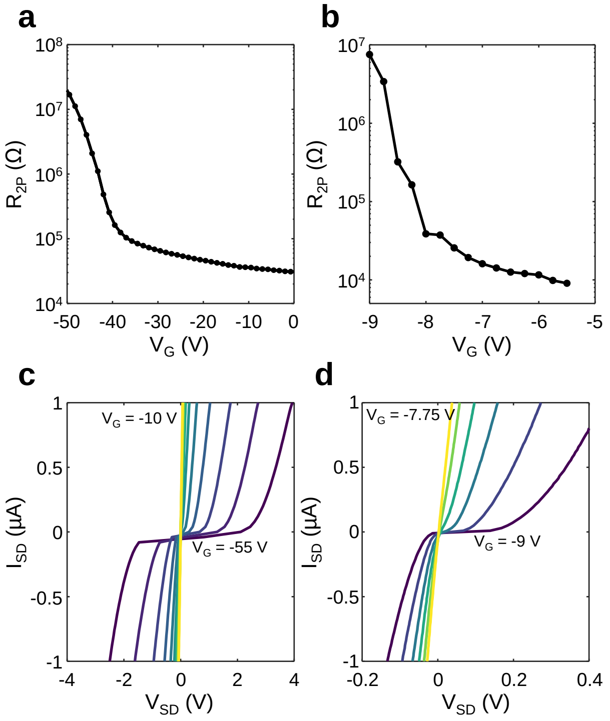


**Figure S2. Transport properties of the MIT in samples C1 and C3. a,b,** zero-bias two terminal resistance of samples C1 (a) and C3 (b), as a function of gate voltage. Both samples show a sharp resistance increase below a threshold gate voltage. **c,d,** IV curves of samples C1 (c) and C3 (d), obtained for various $V_G$. Non-linear IV characteristics onset below the threshold voltages for the resistance increase. These data indicate the onset of an insulating phase at low VG.

## Supplementary Note 4: Pattern of sample C1

Figure S3 shows an optical microscope image of the pattern studied in sample C1 (Figure 2 of the main text). The sample was patterned into a channel of continuously varying width. The area imaged in this experiment had widths between 40 μm and 60 μm.

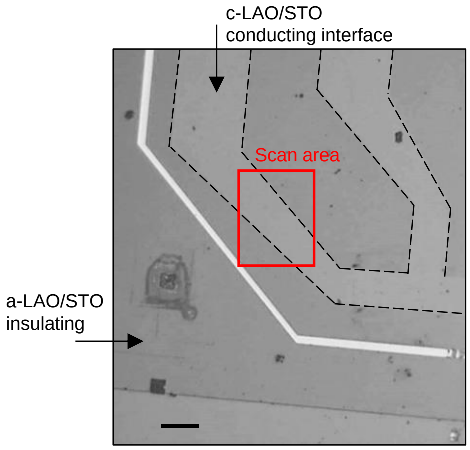


**Figure S3. Optical microscope image of sample C1.** The bright (dark) areas are crystalline (amorphous) LAO. The interface below the crystalline layer is conducting. The dashed lines indicate the edges of the conducting pattern,

the red rectangle indicates the scan area of Figure 2 in the main text. Within the scan area, the device width changes from 40 µm to 60 µm. Scale bar, 50 µm.

## Supplementary Note 5: Distribution of domain boundaries

Figure S1 demonstrates how the domain wall distribution can vary significantly within a single device: the images reveal large mono-domain areas, neighboring regions with a higher density of walls. This is a common feature in LAO/STO devices. Figure S4 shows another example of the large spatial variations in the domain patterns. Within a single device, (field of view: 250 µm × 250 µm), we observed regions with a low density of boundaries, oriented along the [010] direction (rectangle b), large mono-domain regions (rectangle c), high density of [010] boundaries (rectangle d), and [100] boundaries (rectangle e). The resulting response to the gate, shown in Figures S2b-e shows the range of behaviors discussed in the main text: current focusing along domain walls oriented parallel to the overall direction of the flow, percolation in mono-domain regions, and percolation between domain walls oriented perpendicular to the overall direction of the flow.

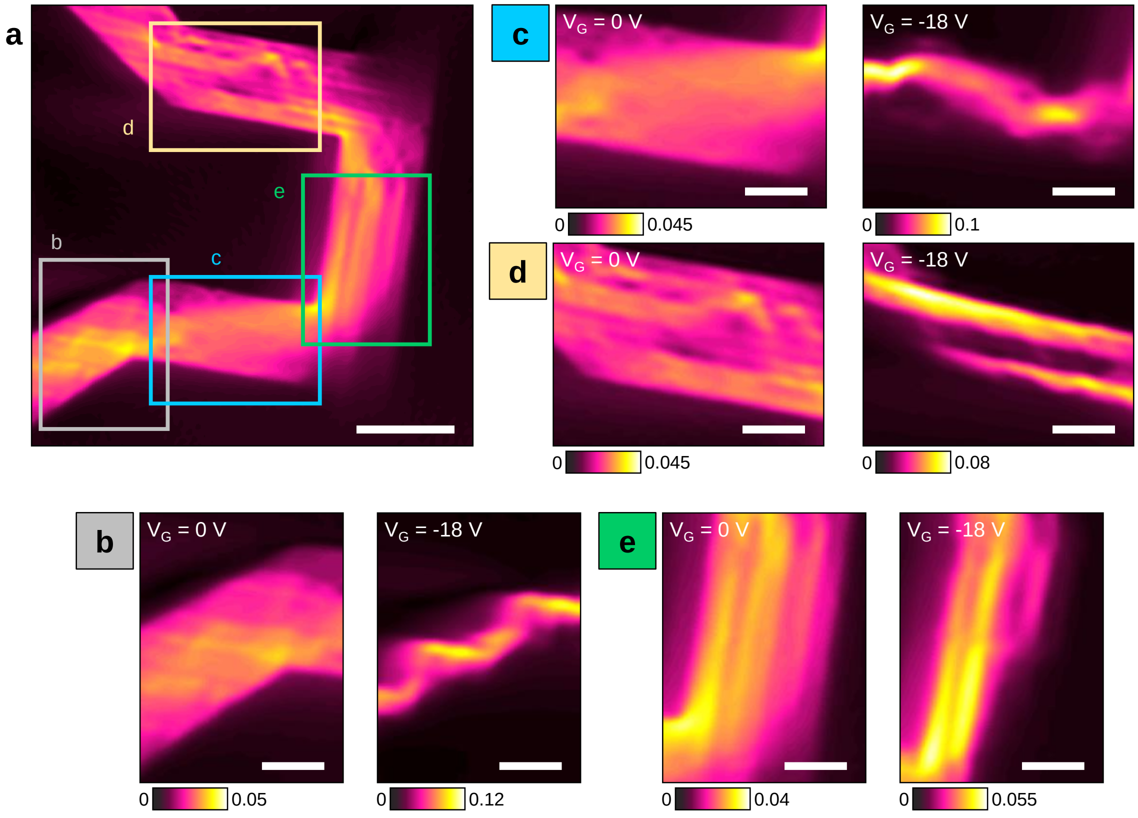


**Figure S4. Current paths near the MIT for various domain patterns on a single device. a,** Large area current density image of a device in sample C3, showing various domain patterns. Scale bar, 50 µm. **b-e,** current density maps of the rectangles shown on panel a, at $V_G$ = 0 V (left) and $V_G$ = -18 V (right), showing the different current paths near the MIT, due to the different domain patterns. Scale bars, 25 µm.

## Supplementary Note 6: Estimation of the gas parameter

Here, we estimate the gas parameter, $r_s$, of LAO/STO, quantifying the ratio between the potential and kinetic energy in the system. For a 2D system, $r_s$ is given by $r_s = 1/\sqrt{\pi n (a_B^*)^2}$, where $n$ is the charge carrier density, and $a_B^* = \hbar^2 \epsilon / m^* e^2$ is the effective Bohr radius. The carrier density[5] (~$1\times10^{13}$ $cm^{-2}$) and the effective mass[8,9] (~$3m_e$) have been measured. We are left with estimating the dielectric constant $\epsilon$, which controls the screening of the electron-electron interactions. Although the dielectric constant of bulk STO at low temperatures is extremely large[10] (~$2\times10^4$), its value close to the 2DEG is significantly lower, since the large electric field generated by the polar LAO layer strongly pins the optical phonon distortion[11]. As a result, an estimate of a surface dielectric constant $\epsilon \sim 100$ is appropriate[12]. The resulting Bohr radius is $a_B^* \cong 2\ nm$, leading to $r_s \cong 1$.

## Supplementary Note 7: RRN results for complex domain patterns

In unpatterned devices, structural domains tend to form strongly correlated patterns, due to the long ranged strain fields they induce. In the main text, we investigated through experiment and simulations an example of one such pattern: an array of elongated, equally spaced domain walls. Here, we used the RRN simulations to consider more complex patterns, which had been observed in previous optical studies[13]. These patterns contain arrays of intersecting boundaries of various orientations and irregular length and spacing (Figure S5a-c). For all patterns we studied, the critical point was shifted downwards with increasing network size, but the rate of change varied considerably between the patterns (Figure S5g).

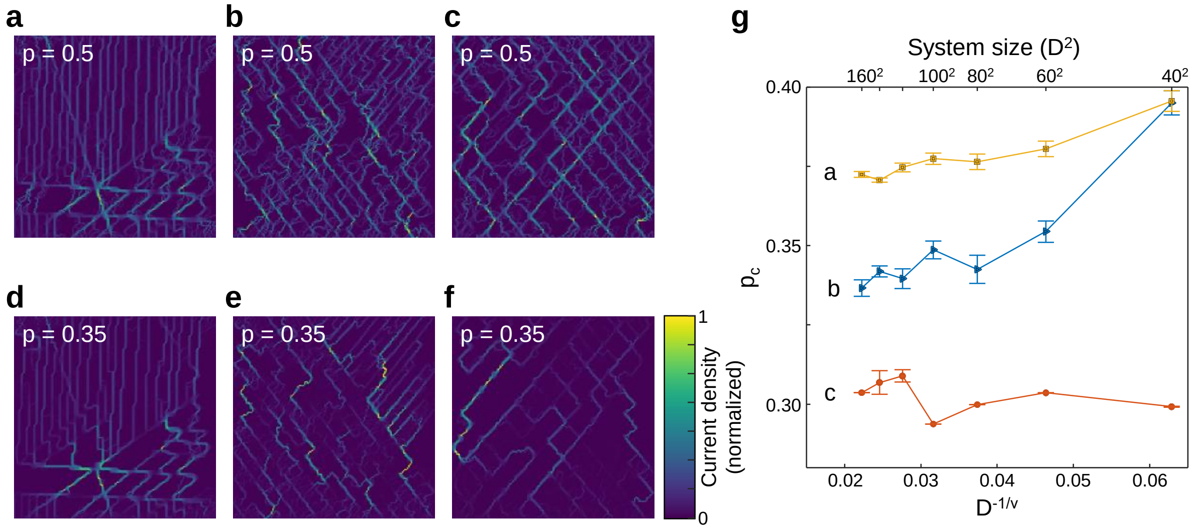


**Figure S5. RRN simulations for complex domain patterns. a-c**, current distributions from single realizations of the models, for various domain patterns which appeared in unpatented samples[13], at p = 0.5. **d-f**, same as panels a-c, but for p = 0.35. **g**, Size dependence of the shifted critical point for each configuration.

## Supplementary Note 8: Estimation of the shift to the critical point

Here, we provide a scaling argument which explains the size-dependent shift of the critical point, due to the introduction of conducting channels. We first consider the case of domain boundaries whose position and size is randomly distributed, with some typical separation $l$. In this case, the transition occurs when the correlation length is of the order $l$,

$$\xi = |p - p_c|^{-\nu} \cong l, \tag{2}$$

suggesting a shift to the critical point

$$p = p_c - (1/l)^{1/\nu}, \tag{3}$$

which depends on the spacing $l$ of the walls. Experimentally, such domain wall distributions can be realized in magnetic materials, where an external magnetic field can be used to change the typical size of domains. The distribution of structural domain walls, however, is often strongly correlated. These walls tend to form highly correlated distributions, which are elongated along the crystallographic direction. For this type of distributions, the critical concentration strongly depends on the details of the domain pattern. If the walls are parallel to the overall direction of the current flow, the transition is suppressed altogether. On the other hand, for equally spaced walls perpendicular to the flow, the , the critical value of $p_c$ is pushed to a size-dependent value, $p_{DW}$: when the length of the walls is comparable to the system size, $D$, the probability to find a percolating path, $P \sim De^{-l/\xi}$, is no longer exponentially small in $D$. The transition is therefore shifted to a lower value, logarithmically dependent on $D$,

$$p_{DW}(D, l) \sim p_c \;\; - \left[\frac{\log D}{l}\right]^{\frac{1}{\nu}}. \tag{4}$$

## Supplementary Note 9: Domain boundaries perpendicular to the overall direction of the current

In the metallic regime, the geometry of the device controls the direction of the current flow. The perturbation from domain boundaries depends on their orientation with respect to the device geometry: boundaries oriented along the device (such as those in Figure S4d,e) can draw larger portions of the current than boundaries oriented perpendicular to the overall direction of the current (such as Figure S4b).

We explain this behavior in terms of random resistor networks. First, consider a network with homogeneous conductivity ($p = 1$), where the voltage gradient is applied in the y direction. In this case, the least resistive path between a point at the bottom edge, and the top edge of the network, is the same as the shortest path. The shortest path is a straight line in the y direction, so no current will flow in the x direction. Adding a line of less resistive nodes in the x direction does not change this behavior, because flow in the x direction elongates the path, making it more resistive.

At a finite disorder ($p < 1$), the least resistance path is no longer the shortest path. The path becomes longer because it is favorable to avoid highly resistive nodes. Flow along a perpendicular conductive channel becomes more likely, because nodes immediately before or after the channel may be insulating. Close to the percolation threshold, the modulation is largest, because the highly conducting channels have a high probability of connecting dangling bonds (parts of the percolating cluster with a dead end that does not connect to the electrodes) to the backbone. This significantly modifies the current flow, because it generates several paths connected in parallel, as opposed to a single series path.

## Supplementary Note 10: Survey of observed variations in transport at low carrier concentrations

The non-universal current backbone, and size and geometry dependence of the critical point imply that transport behavior of mesoscopic samples near the MIT must show strong variations between samples. There is a large volume of work gate-tunable properties of LAO/STO, which supports this conclusion. The percolation-type MIT observed through conductivity measurements [5,6] showed large variations in the critical carrier density between different samples[5], and quadratic magneto-resistance near the transition, inconsistent with the linear behavior expected for percolation in an "ordinary" disordered system[6]. Further, there are multiple conflicting reports about the gate tunable magnetoresistance and Shubnikov-de-Hass oscillations (see ref.[14] for a review). The large variability between studies strongly suggests that the source of inconsistencies is intrinsic to the material system.

Similarly, our results offer a new perspective on the MIT in bulk, three dimensional STO. Even though the experimentally observed Fermi surface of doped crystals[15] is in good agreement with its calculated band structure[16], bulk STO do not display a sharp MIT, and the dilute metal gradually fades away as the doping is reduced[17]. While this idea requires further experimental effort, our results identify domain boundaries as a key player in the emergent metallicity near the putative critical doping, in both bulk STO, and its 2D counterpart.

## References


1. Bell, C., Harashima, S., Hikita, Y. & Hwang, H. Y. Thickness dependence of the mobility at the $LaAlO_3/SrTiO_3$ interface. *Appl. Phys. Lett.* **94**, (2009).
2. Nowack, K. C. *et al.* Imaging currents in HgTe quantum wells in the quantum spin Hall regime. *Nat. Mater.* **12**, 787 (2013).
3. Roth, B. J., Sepulveda, N. G. & Wikswo, J. P. Using a magnetometer to image a two-dimensional current distribution. *J. Appl. Phys.* **65**, 361–372 (1989).
4. Spivak, B., Kravchenko, S. V, Kivelson, S. A. & Gao, X. P. A. Colloquium: Transport in strongly correlated two dimensional electron fluids. *Rev. Mod. Phys.* **82**, 1743–1766 (2010).
5. Liao, Y. C., Kopp, T., Richter, C., Rosch, A. & Mannhart, J. Metal-insulator transition of the $LaAlO_3$-$SrTiO_3$ interface electron system. *Phys. Rev. B Condens. Matter* **83**, 75402 (2011).
6. Lin, W.-N. *et al.* Electrostatic Modulation of $LaAlO_3/SrTiO_3$ Interface Transport in an Electric Double-Layer Transistor. *Adv. Mater. Interfaces* **1**, 1300001 (2014).
7. Caviglia, A. D. *et al.* Electric field control of the $LaAlO_3/SrTiO_3$ interface ground state. *Nature* **456**, 624–627 (2008).
8. Dubroka, A. *et al.* Dynamical Response and Confinement of the Electrons at the $LaAlO_3/SrTiO_3$ Interface. *Phys. Rev. Lett.* **104**, 156807 (2010).
9. Collignon, C., Lin, X., Rischau, C. W., Fauqué, B. & Behnia, K. Metallicity and Superconductivity in Doped Strontium Titanate. *Annu. Rev. Condens. Matter Phys.* **10**, 25–44 (2019).
10. Sakudo, T. & Unoki, H. Dielectric Properties of $SrTiO_3$ at Low Temperatures. *Phys. Rev. Lett.* **26**, 851 (1971).
11. Worlock, J. M. & Fleury, P. A. Electric field dependence of optical-phonon frequencies. *Phys. Rev. Lett.* **19**, 1176 (1967).
12. Copie, O. *et al.* Towards Two-Dimensional Metallic Behavior at $LaAlO_3/SrTiO_3$ Interfaces. *Phys. Rev. Lett.* **102**, 216804 (2009).
13. Erlich, Z. *et al.* Optical Study of Tetragonal Domains in $LaAlO_3/SrTiO_3$. *J. Supercond. Nov. Magn.* **28**, 1017–1020 (2015).
14. Pai, Y.-Y., Tylan-Tyler, A., Irvin, P. & Levy, J. Physics of $SrTiO_3$-based heterostructures and nanostructures: a review. *Reports Prog. Phys.* **81**, 36503 (2018).

15. Lin, X. *et al.* Critical Doping for the Onset of a Two-Band Superconducting Ground State in $SrTiO_{3-\delta}$. *Phys. Rev. Lett.* **112**, 207002 (2014).
16. van der Marel, D., van Mechelen, J. L. M. & Mazin, I. I. Common Fermi-liquid origin of $T^2$ resistivity and superconductivity in n-type $SrTiO_3$. *Phys. Rev. B* **84**, 205111 (2011).
17. Spinelli, A., Torija, M. A., Liu, C., Jan, C. & Leighton, C. Electronic transport in doped $SrTiO_3$: Conduction mechanisms and potential applications. *Phys. Rev. B* **81**, 155110 (2010).
